# Inadequate Hand Washing, Lack of Clean Drinking Water and Latrines as Major Determinants of Cholera Outbreak in Somali Region, Ethiopia in 2019

**DOI:** 10.3389/fpubh.2022.845057

**Published:** 2022-05-06

**Authors:** Jemal Mussa Challa, Tamirat Getachew, Adera Debella, Melkamu Merid, Genanaw Atnafe, Addis Eyeberu, Abdi Birhanu, Lemma Demissie Regassa

**Affiliations:** ^1^School of Public Health, College of Health and Medical Sciences, Haramaya University, Harar, Ethiopia; ^2^School of Nursing and Midwifery, College of Health and Medical Sciences, Haramaya University, Harar, Ethiopia

**Keywords:** cholera, outbreaks, risk factors, acute watery diarrhea, infection

## Abstract

**Introduction:**

Cholera remains a serious public health problem characterized by a large disease burden, frequent outbreaks, persistent endemicity, and high mortality, particularly in tropical and subtropical low-income countries including Ethiopia. The recent cholera outbreak in the Somali region began on 4 September to 1 November 2019. Cholera may spread rapidly through a population so that an early detection and reporting of the cases is mandatory. This study aimed to identify determinants of cholera infection among >5 years of age population in Somali region, Ethiopia.

**Methods:**

A community-based unmatched case-control study was conducted among 228 (76 cases and 152 controls, 1:2 ratio) systematically selected population. Data were collected using a structured questionnaire administered by an interviewer and a record review. Descriptive statistics and multivariable logistic regression analysis was used to identify the determinants of the risk factors of cholera infection with a 95% confidence interval and statistical significance was declared a tap-value < 0.05.

**Results:**

A total of 228 participants (33.3% cases and 66.7% controls) were enrolled in this study. The majority of the cases were in the range of 20–49 years of age (69.7%). The odds of acquiring cholera infection increased significantly by drinking unsafe pipe water (AOR 4.3, 95% CI 1.65–11.2), not having a household level toilet/latrine (AOR 3.25, 95% CI 1.57–6.76), hand washing only sometimes after the toilet (AOR 3.04, 95% CI 1.58–5.86) and not using water purification methods (AOR 2.3, 95% CI 1.13–4.54).

**Conclusion:**

Major risk factors for cholera infection were related to drinking water and latrine hygiene. Improvement in awareness creation about cholera prevention and control methods, including water treatment, hygiene and sanitation were crucial in combating this cholera outbreak. Primary public health actions are ensuring clean drinking water, delivery of water purification tablets, soap and hand sanitizers and provision of health care and outbreak response. Long term goals in cholera affected areas include comprehensive water and sanitation strategies. Overall, the strategic role of a multi-sectoral approach in the design and implementation of public health interventions aimed at preventing and controlling cholera are essential to avert cholera outbreaks. Preparedness should be highlighted in cholera prone areas like Somali region especially after drought periods.

## Introduction

Cholera is an acute watery diarrheal disease caused by ingestion of food or water contaminated with toxigenic strains of *Vibrio cholerae*, serogroups O1 or O139 (1). Cholera is an extremely severe illness causing predominantly severe acute watery diarrhea. Incubation period varies between 12 h and 5 days for a person to show symptoms after ingesting contaminated food or water or contact to an infected individual (2). Although the majority of cases are asymptomatic or with mild symptoms, cholera infection may cause severe diarrhea and vomiting, and in very severe cases extreme dehydration, metabolic acidosis, and even death (3). Global estimates of cholera cases and deaths are about 2.9 million and 95,000 per year, respectively (4), disproportionately affecting sub-Saharan African countries, especially since the onset of the seventh cholera pandemic in 1961 (1). For instance, 17 African countries reported over 150,000 cholera cases from all outbreaks in 2017 (5).

Humans are the only known natural hosts of *Vibrio cholerae*. The global epidemiology of cholera presents the disease occuring in tropical and subtropical, low-income regions of the world with inadequate food and water hygiene (predominantly Sub-Saharan Africa, Asia, and Latin America). Imported cases elsewhere occasionally occur among travelers returning from the endemic areas (6).

The global burden of cholera is largely unknown because majority of cases remain unreported. The low reporting may be attributed to the limited capacity of epidemiological surveillance and laboratory capacity, as well as social, political, and economic disincentives for reporting. Altogether about 1.3 billion of global population are estimated to be at risk of cholera in endemic countries. An estimated 2.86 million cases of cholera occur annually in endemic countries. Among these cases, there were an estimated 95,000 deaths in year 2008–2012 (4).

African countries reported 46% (3,221,050) of all globally reported suspected cholera cases to the World Health Organization (WHO) during 1970–2011 (7). The number of African countries with indigenous cholera cases increased from 24 in 1971 to 30 in 1998 peaking at 36 in 2008 of all 46 African countries. Sub-Saharan Africa had the highest burden of cholera cases globally with 86% of reported cases between 1970 and 2011 (7). Although there has been a decrease in the endemicity and intensity of epidemics across the continent, the case fatality rates remain higher in Africa (at around 60%) than in Asia (29%) (4, 8). Heavy rainfall and flooding are known significant risk factors for cholera outbreaks (9). Drought, on the other hand, plays an important role in the primary causes of cholera, which can be explained in a variety of ways. One possible explanation is that drought causes scarcity or shortage of water, causing residents to use water available in their nearby regardless of the water quality, common in Ethiopia and Africa. Drought and poverty may result in socioeconomic challenges in low and middle income countries, resulting in outbreak prone poverty-related diseases like Cholera (10).

History in Ethiopia revealed that the first outbreak of cholera occurred in 1,834 with substantial mortality in the population. Most of the cholera outbreaks in the country have occurred following a drought period (11). A major cholera outbreak occurred in nine regions of Ethiopia between January and December 2017 (peaking in April-May, 2017) with 48,814 cases and 880 deaths (CFR 1.8%). Cases were predominantly from the Somali region (89% cases and 96% deaths respectively) (12). The severe clean and overall water shortage propelled the cholera outbreak increasing the risk of Severe Acute Malnutrition among children (12, 13). Currently, acute watery diarrhea problems are becoming the leading health-disruptive events in Ethiopia. The outbreak under study was of smaller scale with 110 cases occurring in the same area of Somali region. Due to the region's remoteness, the high proportion of nomadic lifestyles, and incomplete record keeping, the case and death numbers are often inadequately recorded and risk factors not well characterized. This study identified determinant factors of cholera infection in the household level, once again highlighting the importance of water and sanitation hygiene against this devastating pathogen. Moreover, we highly encourage to strengthen disease surveillance and national preparedness to rapidly detect and respond to these outbreaks.

## Methods and Materials

### Study Area and Period

This study was carried out in the Erer district located in the Sitti zone of the Somali region, eastern Ethiopia. It was found at 270 km to northwest from the Regional Health Bureau found in Jigjiga city (regional capital). According to the recent population projection, the total population of Erer district was 69,129 in 2019 of which 11,714 (17%) were urban inhabitants and 57,415 (83%) were pastoralists, whose life relies on livestock. The health care system manages and coordinates the operation of the primary health care services, which currently consists of 4 health centers and 18 health posts. The overall health service coverage of the district for Health Center and Health Post is 86.7 and 74%, respectively (14). Erer district has neither all-weather gravel roads nor community roads; only 16.51% of the total population has access to clean drinking water (14). The data were collected between June and July 2020 in Erer district, Somalia's regional state, Ethiopia.

### Study Design and Population

A community-based case-control study was designed and employed. The study described the outbreak in person and over time using descriptive epidemiology and statistics to generate hypotheses about possible exposures that were common to the cases. Populations who were 5 years and older and lived in seven affected Kebeles (the smallest administrative unit in Ethiopia) of Erer district were the source of the population. We excluded children <5 years from the study to increase specificity of the case definition (15) and children were treated if symptomatic. This is common practice with diarrhea prone diseases, acute watery diarrhea among >5 year olds is quite diagnostic of cholera, while in young children, other causes such as rota virus may be involved. Patients over 5 years of age and registered at the health institutions with acute watery diarrhea and their randomly chosen neighbors were enrolled in this study. The first cases occurred in rural kebeles. The cases from urban kebels were enrolled with controls from the same area, while cases from rural also enrolled with controls living in a rural area. All kebeles of the study area are assumed to be similar in water supply coverage and sociodemographic characteristics. Those patients who were under antibiotic treatment for other diseases during the time of data collection were excluded from the study.

### Sample Size and Sampling Procedures

Epi Info version 7.1.1 software was used to estimate the sample size using the double population proportion formula. Controls were recruited randomly by selecting a household by every two households nearby the case household. The following assumptions were considered to estimate the required sample size; 95% confidence level, 80% power, surface water contact as a risk factor with the lowest odds ratio of 2.3 (16), the proportion of controls with exposure 40% and odds ratio of 2.3. The final estimated sample size by assuming of 10% non-response rate was 228 with 76 cases and 152 controls. The case register logbooks were reviewed for morbidity and mortality reports from September 4 to November 1, 2019, in the district health offices and health facilities. Cases were patients diagnosed with cholera disease and controls were randomly selected individuals from the same Kebele where the cases lived or neighborhood people without cholera disease.

### Data Collection Procedures and Instrument

Data were obtained by reviewing cases and morbidity registry logbooks and all 228 cases were reviewed using a structured pre-tested questionnaire adapted from previous studies (14–16). Data were collected by three trained diploma clinical nurses and one public health officer under the supervision of two MSc clinical nurses. Data on demographic information and related risk factors including food hygiene, hand washing, access to water, hygiene and sanitation, and exposure factors were also collected.

### Operational Definition

**Case**: Any person who was 5 years of age or more with profuse acute watery diarrhea and vomiting for whom their stool has been detected with Vibrio cholerae O1 or O139 (17).

**Control:** Control was an individual older than 5 years and lived in the same kebele as the case and did not have any clinical symptoms suggestive of cholera or diagnosed with cholera during the outbreak period, from September 4 to November 1, 2019 (18).

**A suspected case** was the one who aged 5 years or more developed acute watery diarrhea, with or without vomiting but not confirmed for Vibrio cholera (17).

### Data Quality Assurance

To ensure the quality of the data, the questionnaire was prepared in English language and translated to the local language (Affi Somali) for data collection and then retranslated back into English to ensure the consistency of the data. The training was given for data collectors on data collecting tools and data collection procedures. Then the questionnaire was pre-tested on 5% of the total sample size at nearby communities 1 week before the actual data collection period to ensure its validity. Furthermore, the primary investigator and supervisors reviewed and checked the data for completeness and consistency on a daily basis. The data were entered by two data clerks, and the consistency of the entered data was cross-checked by comparing the two separately entered data on EPI-Data software.

### Methods of Data Analysis

Data were entered into Epi Data version 3.1 statistical software while SPSS version 22 was utilized for data analysis. Before analysis, data were cleaned and edited. Descriptive statistics were used to characterize the frequency and proportions. Variables with a *p*-value of ≤0.3 in the bivariable analysis were enrolled into a multivariable logistic regression to identify the predictors. The Hosmer-Lemeshow test was used to check the goodness of the model. Multicollinearity was assessed by a variance inflation factor (VIF). Finally, multivariable logistic regression analysis was carried out to evaluate the combined effect of determinant factors of cholera outbreaks after adjusting for confounding variables. Adjusted odds ratios (AOR) with 95% CI, was used to express the effect of each category on the outcome relative to the reference category. A statistical significance was declared at a *P*-value of < 0.05.

### Ethical Consideration

Ethical clearance was secured from Haramaya University College of Health and Medical Sciences Institutional Health Research Ethics Review Committee. Before data collection, ethical clearance and a letter of cooperation were obtained from the University to the Erer district. Participants and Caretakers were informed accordingly about the purpose of the study and the importance of their participation. Informed, voluntary, written and signed consent was taken from all participants aged above 18 years old and parents/caretakers for those whose age was below 18 years. The right to refuse, privacy, and confidentiality were kept throughout the data extraction. Furthermore, mothers having a sick child with signs and symptoms of diarrhea were counseled to visit the nearby health facility, and also all study participants having under-five children were given health education on childhood illness and cholera disease.

## Results

### Socio-Demographic Characteristics

The outbreak began on 4th September to 1st November 2019. Altogether 228 study participants were recruited for the case-control study of which 76 were cases (who had cholera) and 152 controls (who had no diarrhoeal like illness suggesting cholera). Among 228 study participants, there were slightly more men were more affected compared to women, but the difference was not statistically significant, there were 51.3% (39) cases among men, and 47.5% (37) among women, “*p*-value 0.75”. The age group 20–34 years had a slightly high number of cases 29 (38.2%), while the lowest number of cases were in the age group >50 years 11 (14.5%). The age ranged from 5 to 85 years with a mean age of 33.4 (SD = ±16.6) and 32.9(SD = ±11.3) years for cases and controls, respectively. All the respondents reported to be were Muslims (100%). Farmer was the most reported predominant occupation with 18 (24.7%) of cases and 56 (49.3%) of controls. About 86 (56.6%) of controls and 44 (57.9%) of cases had average monthly income of 1,000–1,900 Ethiopian birr (Table 1).

**Table 1:** -demographic characteristics of study population, 2019.

**Variables**	**Categories**	**Cholera status**	
		**Case (%)**	**Control (%)**
Age	5–19	12(15.8)	11(7.2)
	20–34	29(38.2)	72(47.4)
	35–49	24(31.6)	56(36.8)
	≥50	11(14.5)	13(8.6)
Sex	Male	39(51.3.)	81(53.3)
	Female	37(48.7)	71(46.7)
Marital	Married	52(68.4)	112(73.3)
	Single	16(21.1)	26(17.1)
	Others	8(10.5)	14(9.2)
Ethnicity	Somali	61(80.3)	126(82.9)
	Oromo	15(19.7)	26(17.1)
Educational status	Illiterate	40(52.6)	78(51.3)
	Read and write	7(9.2)	12(7.9)
	Primary	16(21.1)	41(27.0)
	Secondary and above	13(17.1)	21(13.8)
Occupation	Farmer	18(23.7)	56(36.8)
	Housewife	15(19.7)	22(14.5)
	Governmental employee	5(6.6)	8(5.3)
	Private employee	6(7.9)	9(5.9)
	Merchant	7(9.2)	9(5.9)
	Student	7(9.2)	21(13.8)
	Daily labor	8(10.5)	12(7.9)
	Others	10(13.2)	15(9.9)
Income	500–900	10(13.2)	8(5.3)
	1,000–1,900	44(57.9)	86(56.6)
	2,000–2,900	9(11.8)	32(21.1)
	≥3,000	13(17.1)	26(17.1)

### Descriptions of Cases Using Epidemic Curve

**Figure 1:**
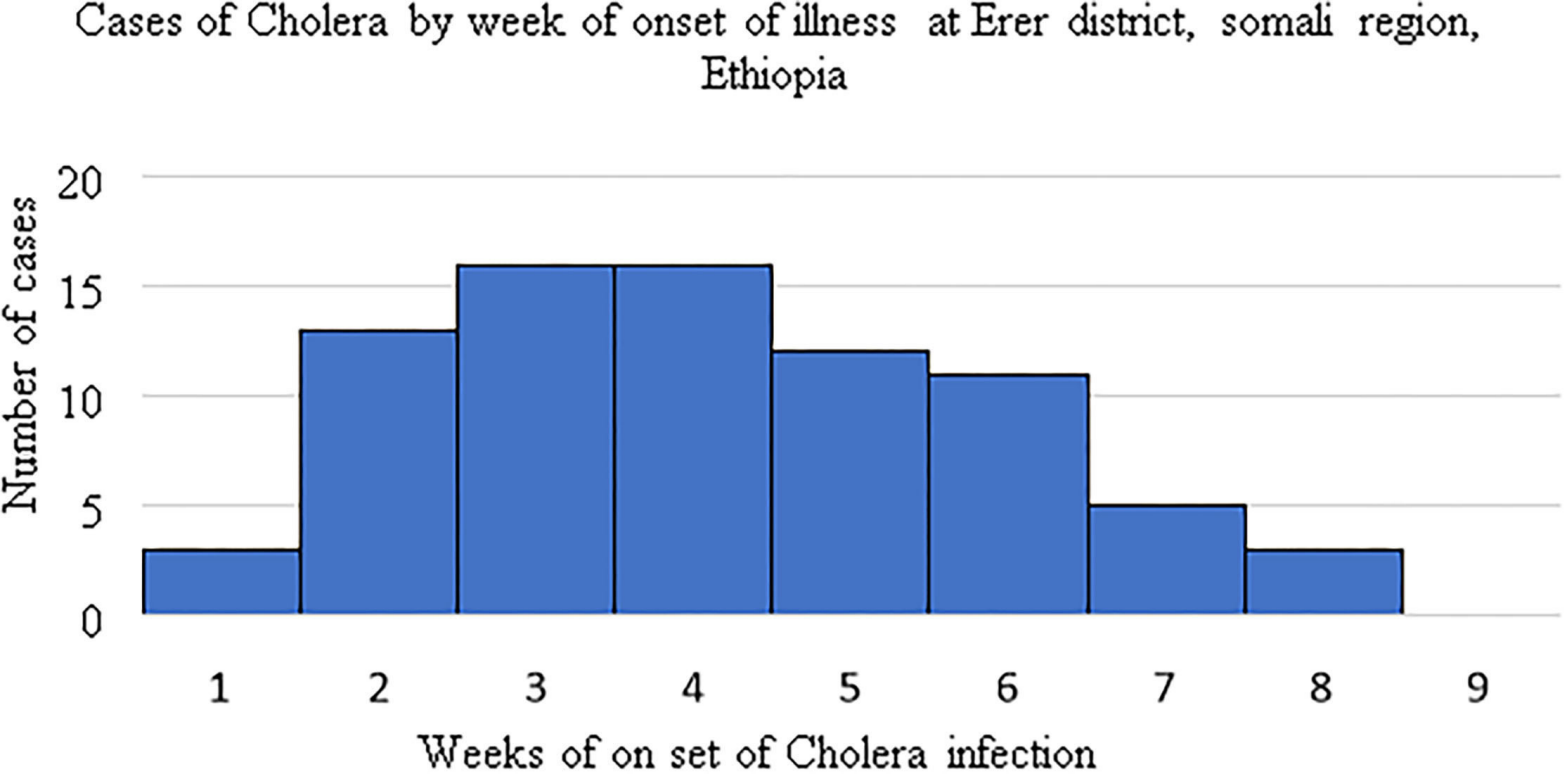
Cholera casesby week of onset of illness, Erer district, Somali region, Ethiopia: 4th September to 1st November 2019.

During the study period the highest cholera cases were recorded in the third and forth weeks 16(21%) each (Figure 1).

**Figure 2:**
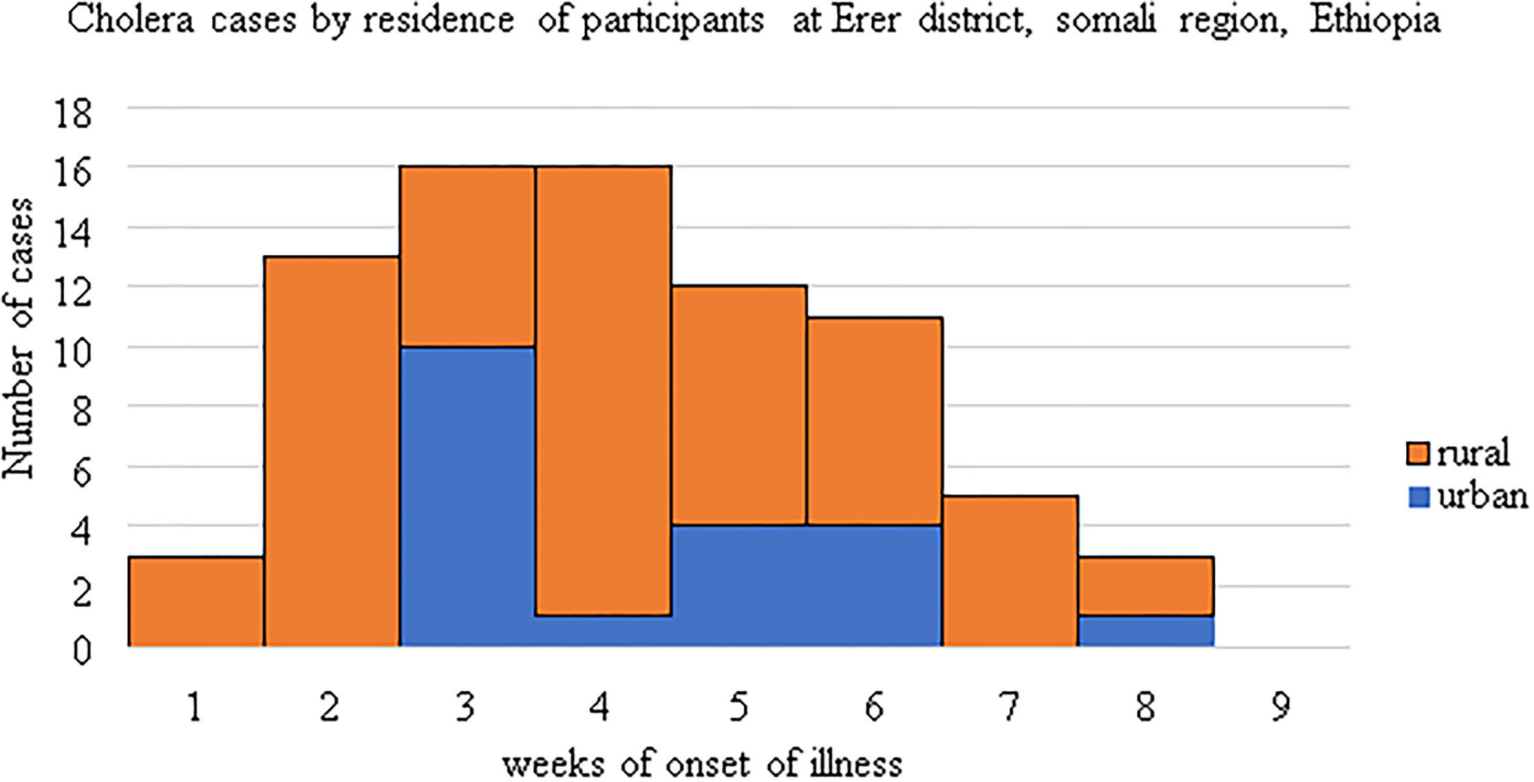
An epidemic curve showing the difference in the distribution of cases by residence and onset weeks of cholera illness.

The first outbreak was evolved from rural kebeles and almost for the 2 weeks almost all cases were reported from rural kebeles, Epidemic curve (Figure 2).

**Figure 3:**
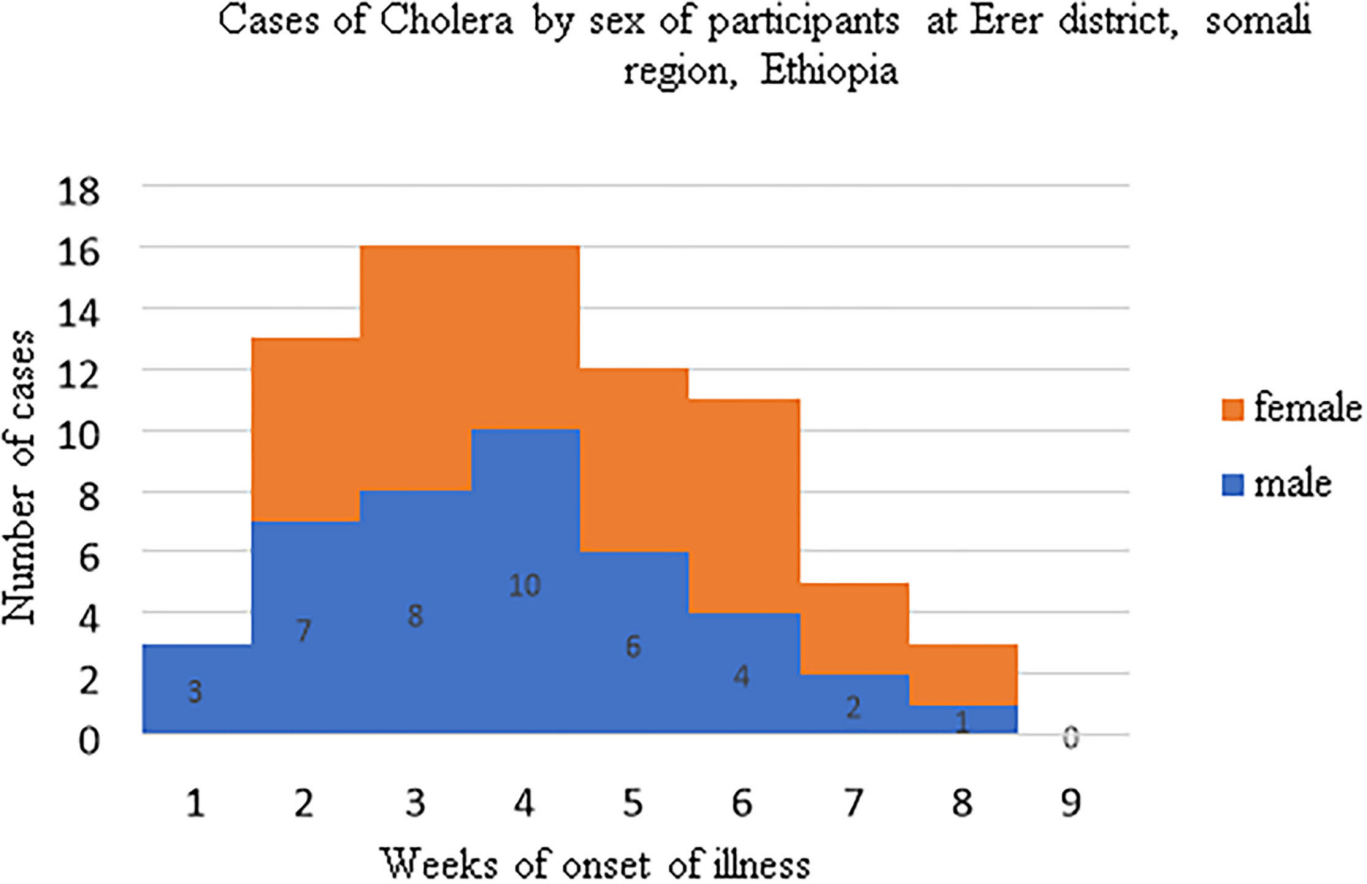
Epicurve showing the distribution of cholera illness based on the sex of participants.

During the study period the first three cases were identified among males. Starting from the second week males and females were almost equally attacked by cholera outbreak (Figure 3).

### Awareness Toward Cholera

Of the total of study participants, 48 (63.8%) of the cases had information about cholera, of which 17 (22.4%) cases and 31 (20.4%) controls believed that drinking contaminated water would increase the transmission of cholera. From those 118 (51.8%) who had information about cholera, 37 (48.7%) cases and 52 (34.2%) controls responded as cholera was a preventable disease. Respondents were asked about what was the first place to go for treatment of cholera disease, 12 (15.8%) cases and 24 (15.8%) controls reported as they were going to the health facility to get treatment for cholera (Table 2).

**Table 2:** Knowledge of cholera and care-seeking habit in Somali region, 2019.

**Variables**	**Categories**	**Cholera status**	
		**Cases (%)**	**Controls (%)**
Heard about cholera	Yes	48(63.2)	70(46.1)
	No	28(36.8)	82(73.2)
Cholera transmission	Contaminated food	15(19.7)	20(13.2)
methods	Contaminated water	17(22.4)	31(20.4)
	Contact with patient	16(21.1)	19(12.5)
Care seeking during cholera	Go to a health facility	12(15.8)	24(15.8)
attack	Seek traditional healer	10(13.2)	11(7.2)
	Uses ORS	19(25.0)	23(15.1)
	Use holy water	7(9.2)	12 (7.9)
Consider cholera treatment	Yes	39(51.3)	57(37.5)
center as a source of	No	9(11.8)	13(8.6)
transmission			
Cholera is a preventable	Yes	37(48.7)	52(34.2)
disease	No	10(13.2)	18(11.8)
Methods used to prevent	Using toilet	6(7.9)	7(4.6)
cholera	Eating cooked food	13(17.1)	16(10.5)
	Using purified water	8(10.5)	15(9.9)
	Hand washing	11(14.5)	13(8.6)

### Hygiene and Sanitation

In the district, 60 (78.9%) cases and 90 (59.2%) controls had a pit latrines at the household level. Of those who had a latrine, 49 (64.47%) cases and 70 (46%) controls used the latrine properly (the latrine had a proper cover, was clean, and had water and soap). In terms of solid waste disposal, the majority of households (50 (65.78%) cases and 115 (75.65%) controls) dumped their waste in open fields or on the street. Approximately 59 (77.63%) cases and 112 (73.68%) controls reported they are knowledgeable about the health problems associated with waste disposal management. The respondents' hygienic practices were also assessed. As a result, 91 (59.9%) controls and 46 (60.5%) cases were washed their hands with water and soap after using the toilet (Table 3).

**Table 3:** Hygiene and sanitation characteristics in Erer District, 2019.

**Variables**	**Categories**	**Cholera status**	
		**Cases (%)**	**Control (%)**
Have toilet at HH level	Yes	60(78.9)	90(59.2)
	No	16(21.1)	62(40.8)
Currently use toilet	Yes	48(63.2)	69(45.4)
	No	13(17.1)	21(13.8)
Condition of the toilet	Clean	26(34.2)	43(28.3)
(by observation)	Cover slap	10(13.2)	16(10.5)
	Sign of utilization	22(31.6)	30(20.4)
Time to wash hand	After using toilet	46(60.5)	91(59.9)
	After cleansing child	8(10.5)	33(21.7)
	Before preparing food	7(9.2)	18(11.8)
	Before feeding child	15(19.7)	10(6.6)
Frequency of hand	Sometimes	109(71.7)	43(28.3)
washing after toilet	Always	39(51.3)	37(48.3)
Frequency of	Always	52(68.4)	103(67.8)
handwashing after	Sometimes	14(18.4)	29(19.1)
feeding your child	I do not have children	20(13.2)	10(13)

### Source of Water

Regarding the use of water sources, 46 (60.5%) cases and 81 (53.3%) controls used river water, and 11 (14.5%) cases and 35 (23.0%) controls used pipeline water supply. Regarding the water storage of households, 48 (63.2%) cases and 79 (52.0%) controls use jerrican, 15 (19.7%) cases and 54 (35.5%) controls use a bucket, and the rest use (tanker, barrels, and small plastic barrels). On the other hand, 36 (47.4%) cases and 74 (48.7%) controls reported that they use the water purification method for drinking water (Table 4).

**Table 4:** Water source of households in Erer District, 2019.

**Variables**	**Categories**	**Cholera status**	
		**Cases (%)**	**Controls (%)**
Source of water for the	Pipeline	10(13.2)	15(9.9)
HH	Spring	10(13.2)	15(9.9)
	Hand-dug well	13(17.1)	14(9.2)
	Deep well	14(18.4)	15(9.9)
	River	29(32.2)	93(61.2)
Type of container used	Jerry cane	48(63.3)	79(52.0)
to store water at home	Bucket	15(19.7)	54(35.5)
	Others	13(17.1)	19(12.3)
Method of water	Pouring	48(63.2)	96(63.2)
fetching from the	Dip with cup	16(21.1)	38(25.0)
container	Others	13(15.8)	18(11.8)
Container has cover/lid	Yes	69(90.8)	137(90.1)
	No	7(9.2)	15(9.9)
Think as the water is	Yes	34(44.7)	77(50.7)
safe	No	42(55.3)	75(49.3)
Purify the water	Yes	21(27.6)	66(43.4)
	No	55(72.4)	86(56.6)
Methods of water	Filtration	8(10.5)	24(15.8)
purification	Sedimentation	5(6.6)	20(13.8)
	Water chemicals	8(10.5)	22(14.5)
Water purification	No	62(81.5)	114(75.0)
chemical is easily	Yes	14(18.5)	38(25.0)
available			

### Eating Habits

**Figure 4:**
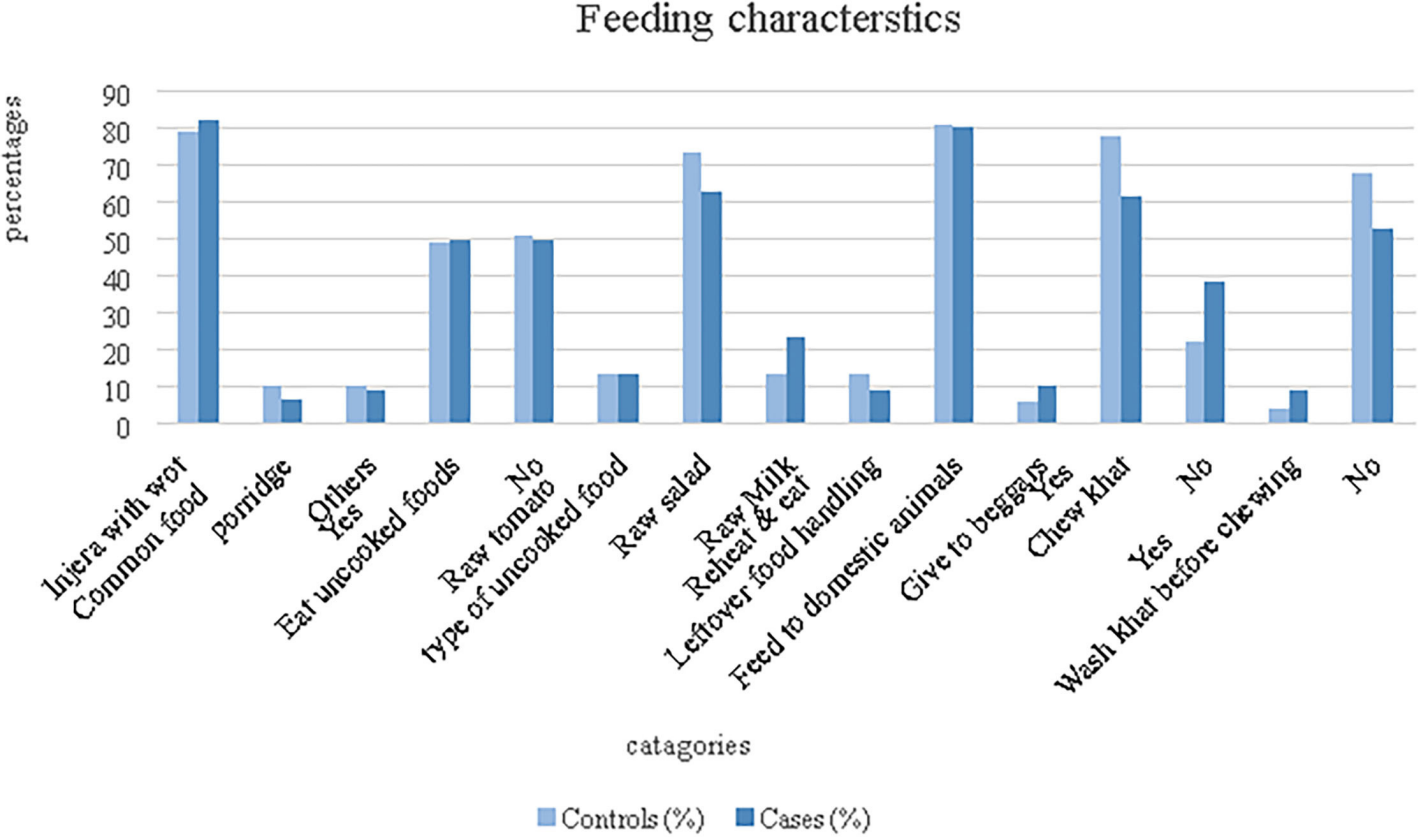
Feeding characteristics of the population in Erer woreda, 2019.

The cultural food in the study area is “*injera”* (a kind of loaves which is prepared from endemic cereal grain “Teff”) with “*wot*” (Stew). Accordingly, 64 (84.21%) cases and 120 (78.94%) controls were reported to be usually eating “*injera*” with “*wot”*. Similarly, most (80.7%) of the population used leftover foods to feed their domestic animals. Moreover, 47 (61.84%) cases and 109 (71.74%) controls were reported as they usually chew khat (a leaf plants chewed traditional as recreational drugs for euphoric purpose and it also has a religious connotation) (Figure 4).

### Determinants of Cholera Outbreak

In the final model, heard about cholera, having toilet at a household level, frequency of handwashing after toilet, source of water, and use of water purifying methods were significantly associated with cholera infection.

The odds of acquiring cholera infection increased significantly by not having information about cholera [AOR = 2.6, 95% CI:(1.36–5.09)], not having a household level toilet/latrine [AOR = 3.25, 95% CI:(1.57–6.70)], hand washing only sometimes after the toilet [AOR = 3.04, 95% CI:(1.58–5.86)], drinking of spring water [AOR = 2.9, 95% CI:(1.04–8.2)], drinking of hand-dug well water [AOR = 3.6, 95% CI (1.3–10.2)], drinking deep well water [AOR = 4.2, 95% CI:(1.56–11.5)], drinking from river sources [AOR = 4.3, 95% CI: (1.65–11.2)], and not using water purification methods [AOR = 2.3, 95% CI:(1.13–4.54)]. Educational level (AOR 1.1, 95% CI 0.44–2.67) and eating uncooked food (AOR 1.4, 95% CI 0.74–2.69) were not significantly associated with cholera infection (Table 5).

**Table 5:** Multivariable analysis of determinants of cholera outbreak among population aged above 5 years in Somali Region, Ethiopia, 2019.

**Variables**	**Categories**	**Cholera status**		**COR (95%C)**	**AOR (95%CI)**
		**Cases**	**Controls**		
Educational status	Illiterate	40(52.6)	78(51.3)	0.8(0.37–1.82)	1.1(0.44–2.67)
	Read and write	7(9.3)	12(7.9)	0.94(0.29–3.0)	1.3(0.32–4.8)
	Primary school	16(21)	41(27)	0.63(0.26–1.55)	0.77(0.27–2.1)
	Secondary school	13(17.1)	21(13.8)	1	1
Heard about Cholera	Yes	48(63.2)	70(46.1)	1	1
	No	28(36.8)	82(53.9)	2.01(1.14–3.5)	2.6(1.36–5.09)^*^
Have toilet at HH level	Yes	60(78.9)	90(59.2)	1	1
	No	16(21.1)	62(40.8)	2.58(1.36–4.89)	3.25(1.57–6.76)^*^
Frequency of handwashing after toilet	Sometimes	109(71.7)	43(28.3)	2.4(1.36–4.25)	**3.04(1.58–5.86)**
	Always	39(51.3)	37(48.3)	1	1
Source of water	Pipe water	10(13.1)	15(9.9)	1	1
	Spring	10(13.1)	15(9.9)	1.01(0.32–3.1)	2.9(1.04–8.2)^*^
	Hand dug well	13(17.1)	14(9.2)	1.39(0.46–4.18)	3.6(1.3–10.2)^*^
	Deep well	14(18.4)	15(9.9)	1.4(0.47–4.13)	4.2(1.56–11.5)^*^
	River	29(38.3)	93(61.1)	0.47(0.19–1.15)	4.3(1.65–11.2)^*^
Use water purifying methods	No	55(72.4)	86(56.6)	2.01(1.11–3.64)	2.3(1.13–4.54)
	Yes	21(27.6)	66(43.4)	1	1
Eating uncooked food	Yes	38(50)	75(49.4)	1.02(0.59–1.78)	1.4(0.74−2.69)
	No	38(50)	77(50.6)	1	1

*1, reference; CI, Confidence Interval; COR, Crude Odds Ratio; AOR, Adjusted Odds ratio*;

* *statistically associated*.

## Discussion

This study identified the determinant factors of the cholera outbreak in the Erer area of Somali region in 2019 with 76 case patients identified. This cholera outbreak was lasted between 4th September to 1st November 2019, age and gender distribution was similar to that of the source population, thus affecting all population groups. The outbreak spread evolved from rural kebeles and propagated into urban areas as the date of case registration demonstrated on the logbook, Epidemic curve (Figure 2). The risk factors of acquiringcholera infection were not having a toilet at the household level, low frequency of handwashing after toilet, poor quality drinking water inadequate use of water purifying methods and not heard about cholera previously.

A total number of 110 cases were reported with 1 death from 4 September to 1 November 2019. The case fatality is at the same level as in a cholera outbreak in Addis Abeba in 2016 among those affected with more than 5 years old (19). Cholera outbreaks typically affect all population with high attack rates (4) and the gender distribution was also equal in our study with 52.3 and 47.7% of the cases were among men and women, respectively. Also, the age distribution of the cases was similar to the source population and the age group 20–49 were mostly affected by this outbreak, 69.7% of the cases were within this age group while 8.7% were within the 5–19 years age group, and 8.3% were above 50 years of age. The largest proportion of any occupational group among cholera cases was (23.7%) farmers while the lower proportion (6.6%) was among governmental employee. This could be a mixed picture, because farmers are more prone to drinking contaminated river water, sharing food on the common plate, eating outside the home, the presence of floods at a drinking water source, and sharing the latrine with other families. On the other hand, population density is smaller in the country side, diminishing the risk of spread of cholera. Additionally, since fruit farming is common in the study area, communities might use the fruits without washing, which could have contributed to this outbreak as has been observed previously (20).

In this study, not having information about cholera was significantly associated with the development of the diseases. This finding is in line with the study conducted in Tanzania (21) which found that the occurrence of cholera was higher among less informed respondents and contrary to the study from Kenya (22) and Cameroon (23) which found that not having information about cholera was not associated with the occurrence of cholera infection. Health education campaigns, adapted to local culture and beliefs, should promote the adoption of appropriate hygiene practices, safe preparation and storage of food, and safe disposal of the feces of children. Community engagement is key to long-term changes in behavior and the control of cholera and should be an integral part of other health promotion initiatives (18).

The lack of access to latrine or toilet at home was a known risk factor for cholera outbreaks; this study also found an association between lack of latrine and being a case. This finding was consistent with the study done in Ethiopia Addis Ababa (19) and Accra (7), highlighting that household toilet facilities were not available in the community, making residents dependent exclusively on poorly maintained public toilets while others resorted to open defecation. As a recommendation, in the event of a future epidemic, the government and other concerned bodies, in collaboration with the community, were expected to build an emergency latrines (24). The low frequency of handwashing after the latrine was significantly associated with the development of cholera in the current study. Those individuals who wash their hands sometimes after the toilet were three times more likely to acquire cholera than those individuals who wash their hands always afte the toilet This finding is consistent with studies done in Zambia (25), Ethiopia (19), Nigeria (26), and systematic review and meta-analysis in less developed countries (16). Proper handwashing reduces person-to-person transmission of cholera and ultimately reduces the force of the outbreak (27). Reversely improper and infrequent handwashing after the toilet increases the risk of transmission of cholera. Actions targeting environmental conditions include the implementation of adapted long-term sustainable WASH solutions to ensure the use of safe water, basic sanitation, and good hygiene practices in cholera hotspots (28).

were found to be more prone to get infected with cholera as compared to their counterparts. This result is consistent with findings from Addis Ababa, Ethiopia (19), Eastern Uganda (29), and Cameroon (23). The sources of water for the rural population are mostly from rivers; hand-dug well, deep well, and springs which are not well protected and hence, unsafe water for households (30). The long-term solution for cholera control lies in economic development through universal access to safe drinking water and adequate sanitation. Crucial cholera epidemic preventive mechanism remains to provide a waste management system that separates waste from the water supply. Moreover, if a cholera outbreak is detected, water purification tablets tother with soap need to be distributed to high risks household by concerned entities (31).

The chance of being infected with cholera was lower among populations who practiced the purification method for drinking water. This finding is inconsistent with the study conducted at Lusaka, Zambia (32). Most households in Erer District do not have safe water sources, the water they used was commonly vulnerable to contamination during transportation and storage. This implies there is a need for sustained health education concerning water treatment and storage because habitual water treatment with chlorine and the provision of safe water can be protective factors to similar outbreaks.

The government need to take some innervations to prevent and respond to future cholera outbreak by systematic assessment and upgrading of water sources in cholera hot spot areas, enhance building of latrines at household level establishment of early warning system to detect cholera outbreaks. Once outbreak has been confirmed, establishment of early warning, alert and response systems (EWARS) (33) to all health care facilities, provision of safe drinking water, provision of latrines, delivery of soap and water purification tablets, community engagement, information campaigns were helpful (28, 33, 34).

Unsafe water sources were also significantly associated with a cholera outbreak. Communities that are not able to get drinking water from pipeline sources The study has some limitations. The data were collected retrospectively and did not cover the total population as case catchment was through health care facilities. The causal relationship is not possible to determine in epidemiological studies like the nature of our study design (case-control). However, we tried to minimize selection bias by recruiting the controls from every two households consequently to the right of the household of the cases where no household member had any signs and symptoms of diarrheal disease within the study period, only recent cases were recruited for the study. We were not able to conduct microbiological water testing because of the constraints of the study budget.

## Conclusion

Independent factors such as not having a toilet at a household level, low frequency of handwashing after toilet, poor quality of source of water, and not using water purifying methods were significantly associated with cholera had significant with cholera. Therefore, improvement in awareness creation about cholera prevention and control methods, water treatment, personal hygiene, and environmental sanitation are crucial interventions to combating the cholera outbreaks. Long term goals in cholera affected areas like Somali region needs to include comprehensive water and sanitation strategies and the government needs to enhance latrine constructions, and consideration of professionally constructed emergency latrines to contain further transmission of the outbreak. Overall, the strategic role of a multi-sectoral approach in the design and implementation of public health interventions aimed at preventing and controlling cholera are essential toavert cholera outbreaks.

## Data Availability Statement

The original contributions presented in the study are included in the article/supplementary material, further can be directed to the corresponding author.

## Ethics Statement

The studies involving human participants were reviewed and approved by Haramaya University, College of Health and Medical Sciences, Institutional Health Research Ethics Review Committee (IHRERC). Written informed consent to participate in this was provided by the participants' legal guardian/next of kin.

## Author Contributions

JC: conceptualization, data collection, investigation, methodology, project administration, writing—original draft, and writing—review and editing. TG: conceptualization, methodology, formal analysis, writing—original draft, and writing—review and editing. AD, MM, AE, and LR: conceptualization, methodology, writing—original draft, and writing—review and editing. GA: data curation, formal analysis, methodology, and writing—review and editing. AB: conceptualization, methodology, and writing—review and editing. Moreover, the co-authors wrote the manuscript. All authors were involved in reading and approving the final manuscript. All authors contributed to the article and approved the submitted version.

## Conflict of Interest

The authors declare that the research was conducted in the absence of any commercial or financial relationships that could be construed as a potential conflict of interest.

## Publisher's Note

All claims expressed in this article are solely those of the authors and do not necessarily represent those of their affiliated organizations, or those of the publisher, the editors and the reviewers. Any product that may be evaluated in this article, or claim that may be made by its manufacturer, is not guaranteed or endorsed by the publisher.

## Abbreviations

COR: crude odds ratio
AOR: adjusted odds ratio
WHO: World Health Organization.
